# Investigation of respiratory disease outbreaks of poultry in Bangladesh using two real-time PCR-based simultaneous detection assays

**DOI:** 10.3389/fvets.2022.1036757

**Published:** 2022-12-13

**Authors:** Rokshana Parvin, Congriev Kumar Kabiraj, Ismail Hossain, Alamgir Hassan, Jahan Ara Begum, Mohammed Nooruzzaman, Md. Taohidul Islam, Emdadul Haque Chowdhury

**Affiliations:** ^1^Department of Pathology, Faculty of Veterinary Science, Bangladesh Agricultural University, Mymensingh, Bangladesh; ^2^Population Medicine and AMR Laboratory, Department of Medicine, Faculty of Veterinary Science, Bangladesh Agricultural University, Mymensingh, Bangladesh

**Keywords:** outbreak investigation, RT-qPCR assays, simultaneous detection, respiratory viral pathogens, mixed infection

## Abstract

For rapid and sensitive pathogen screening from field outbreaks, molecular techniques such as qPCR-based simultaneous detections are efficient. Respiratory diseases are the most detrimental diseases to the poultry industry and need to be addressed because of their major economic losses. In the current study, we have applied two different detection assays: one for simultaneous detection of avian influenza virus (AIV; M gene) and subtyping (H5, N1, H9, N2) using TaqMan probe chemistry (TaqMan multitarget) and another for simultaneous detection of Newcastle disease virus (NDV), infectious bronchitis virus (IBV), and infectious laryngotracheitis virus (ILTV) using SYBR Green chemistry (SYBR Green multitarget). Two individual qPCRs were conducted for the detection of four pathogens. Surveillance of tissue (*n* = 158) and oropharyngeal swab (206) samples from multiple poultry flocks during the years April 2020–July 2022 applying the TaqMan and SYBR Green multitarget qPCRs revealed that 48.9% of samples were positive for respiratory infections, of which 17.2% were positive for NDV, 25.5% were positive for AIV, 9.9% were positive for IBV, and only a single positive (0.3%) for ILTV. Among the AIV, 35% were highly pathogenic subtype H5N1 and 65% were low pathogenic subtype H9N2. Co-infections of 2–3 respiratory viruses were also accurately detected. Respiratory viral pathogens are quite common in Bangladeshi poultry and can be successfully detected using multitarget simultaneous real-time quantitative polymerase chain reaction (RT-qPCR) assays like those adopted in the current study. Increased mass surveillance, along with the molecular characterization of the circulating respiratory viruses, is crucial to control the epidemic and subsequently save the Bangladeshi poultry industry.

## Introduction

The worldwide poultry industry is expanding rapidly in response to the rising demand for animal-derived protein. In Bangladesh, poultry is reared both in the backyard and commercial settings where more than 50% of the households keep chickens and almost 30% of the households rear ducks (http://www.bbs.gov.bd/). The Bangladeshi commercial poultry industry comprises broilers, layers, and slow-growing colored meat-type chickens (Sonali) that produce ~0.2 million metric tons of poultry meat and 5,210 million table eggs per year (1). However, 60–70% of commercial hens are kept in small-scale facilities with limited biosecurity and management methods, which enhances disease susceptibility (2).

Intensification of commercial farming leads to the accumulation of multiple pathogens at a single site, in particular, a wide range of respiratory viral pathogens cause huge economic losses (3). The presence of more than one pathogen in hosts at the same time causes the complexity of diseases (3–8). Globally, poultry production encounters more viral infections compared to infections with other pathogens (9). Among different viral pathogens of poultry, avian influenza virus (AIV), Newcastle disease virus (NDV), infectious bronchitis virus (IBV), and infectious laryngotracheitis virus (ILTV) are the most prevalent and can produce disease independently, concurrently, or in association with bacterial agents and mycoplasmas (8, 10–13). Furthermore, the biosecurity of poultry farms in Bangladesh is quite unsatisfactory, particularly in small- to medium-scale poultry farms (14, 15), allowing pathogens to enter the farms. Early diagnosis of respiratory pathogens can minimize mortality in the infected flocks as well as could suggest effective supportive treatment options. The success of vaccination depends on the genetic background of the circulating strains factor and the selection of broadly protective vaccines against avian viral diseases (16, 17).

Respiratory infections are very common in commercial poultry and duck populations of Bangladesh (18–21). Therefore, rapid detection is required, followed by prevention and control. The current study emphasizes the prevalence of respiratory viruses in poultry and their early, accurate detection. Several laboratory methods such as virus isolation in embryonated eggs and organ cultures and serological tests are being used for decades for detecting and differentiating avian respiratory viral infections (3, 22–24). These methods are time-consuming and laborious, particularly when used separately for a single pathogen (25–27). Nowadays, rapid diagnostic tests have been developed for the detection of either viral nucleic acids or viral antigens, including rapid chromatographic and, molecular techniques (28–33). However, those techniques detect only one specific pathogen at a time. The duplex or multiplex PCR has the ability to amplify and differentiate multiple specific nucleic acids. Many countries have implemented respiratory disease surveillance, and the use of multiplex real-time PCR assays has aided in detecting circulating viruses and outbreaks, as well as estimating disease burden (29–32). Recently, a PCR-based panel assay for the detection of multiple poultry respiratory pathogens has been established using a real-time PCR system in a single reaction with SYBR Green reagent (3). A mini version of the Riems Influenza a Typing Array (RITA) assay (34, 35) has also been used in the subtype detection of AIVs and identification of other avian viral pathogens in Bangladesh (20, 36). The RITA assay is very useful for detecting the AIV subtype in a single run, but it is not cost-effective for a country with limited resources. As a result, selecting particular primers and probes for a specific target run may reduce PCR costs while simultaneously enabling rapid diagnosis. The aim of the study was to detect respiratory viral pathogens (AIV, NDV, IBV, and ILT) and selective subtyping of AIVs using two multitarget real-time quantitative polymerase chain reaction (RT-qPCR) simultaneous detection assays from outbreak samples. We adopt previously established assays (34, 35) in a format of multitarget simultaneous runs for the detection of respiratory viral pathogens that are common and endemic in Bangladesh.

## Materials and methods

### Virus strains from repositories

Ten (10) of each positive AIV– subtype H5N1 (*n* = 5) and H9N2 (*n* = 5) along with NDV, IBV, five (5) ILTV, and 10 mycoplasma-positive (referred to as negative here) samples were collected from virus repositories at the Department of Pathology and of which three vaccine strains were purchased from commercial sources (Nobilis ND LaSota, NOBILIS^®^ MA5 + CLONE 30, and Nobilis^®^ ILT, MSD animal health) to adopt and validate the simultaneous RT-qPCR assays.

### Outbreak samples

A total of 364 poultry flocks, including layer (*n* = 97), broiler and broiler breeder (*n* = 115), Sonali (*n* = 63), duck (*n* = 79), pigeon (*n* = 8), and turkey (*n* = 2) with a history of respiratory signs from different commercial poultry flocks in the Mymensingh division of Bangladesh were investigated from April 2020 to June 2022. Dead birds from those natural outbreaks were submitted to the Department of Pathology, necropsy was performed, and gross pathological changes were recorded. A total of 158 tissue samples (lung and trachea from each individual animal were pooled) were collected in sterile tubes. Tissues were homogenized to prepare a 20% w/v suspension in minimum essential medium (MEM, Thermo Fisher Scientific, USA) containing a mixture of streptomycin and penicillin (50 μg/ml), and the supernatant was collected following centrifugation at 3,000 rpm for 10 min. In addition, 206 oropharyngeal swabs were collected from different poultry flocks with a similar history. Five individual birds were randomly chosen, and swabs were pooled in the tube containing MEM and antibiotics and considered single-swab samples. Samples were kept at −70°C until further analysis.

### Nucleic acid extraction and real-time PCRs

Viral DNA or RNA was extracted using a Monarch nucleic acid purification kit (New England Biolabs Inc., USA). One-step RT-qPCR was performed using TaqMan probe-based and SYBR Green chemistry. The current study used target primers developed for specific diseases in previous investigations (28, 35, 37–39), which are listed in Supplementary Table 1.

### TaqMan multitarget RT-qPCR for screening and subtyping of avian influenza virus

The TaqMan probe-based single-step RT-qPCR assay (referred to as “TaqMan multitarget” here) was optimized using a Luna Universal Probe One-Step RT-qPCR Kit (New England Biolabs Inc., USA) and AIV-specific oligonucleotides as used previously (Supplementary Table 1). The final reaction volume was 12.5 μl, containing 2.5 μl of DNA or RNA template, 6 μl of 2× RT-PCR reaction mix, 1 μl of RT- PCR Enzyme Mix, 1 μl of nuclease-free water, and 2 μl of primer probe mix (10 pmol each). The RT-qPCR thermocycling conditions were 55°C for 10 min (reverse transcription) and 95°C for 1 min (initial denaturation), followed by 45 cycles at 95°C for 10 s (denaturation) and 60°C for 30 s (annealing and elongation) with the reading of fluorescence in this step. The Optimized TaqMan Multiplex is used for the simultaneous detection of generic AIV (M gene) and subtype of AIV (H5, H9, N1, and N2 genes).

### SYBR green multitarget RT-qPCR for screening of other respiratory pathogens

The detection of NDV, IBV, and infectious laryngotracheitis (ILT) using single-step RT-qPCR SYBR Green assay (referred to as “SYBR Green multitarget” here) was carried out by specific target primers against avian paramyxovirus 1 NP gene, IBV UTR region, and ILT glycoprotein gene-specific oligonucleotides, respectively (Supplementary Table 1). The assay was carried out using a Luna Universal One-Step RT-qPCR Kit (New England Biolabs Inc., USA) containing SYBR Green reagents following a previously established protocol (23). The final reaction volume was 12.5 μl, including 2.5 μl of the RNA template, 5 μl of 2× RT-PCR SYBR reaction mix, 0.5 μl of Luna WarmStart RT Enzyme Mix, 2.5 μl of nuclease-free water, and 2 μl of primer mix (10 pmol each). The RT-qPCR thermocycling conditions were 55°C for 10 min (reverse transcription) and 95°C for 2 min (initial denaturation), followed by 45 cycles at 95°C for 10 s (denaturation) and 60°C for 30 s (annealing and elongation) with a reading of fluorescence in this step. Immediately after PCR, a melting curve analysis was performed with a continuous temperature increment of 0.5°C/s between 65 and 95°C. Samples yielding cycle threshold (Ct) values ≤ 32 with an appropriate melting temperature (Tm) were considered positive.

### Laboratory validation

In total, 45 reference samples, AIV (*n* = 10), NDV (*n* = 10), IBV (*n* = 10), ILTV (*n* = 5)-positive samples (virus from the repository and vaccine strains), and avian mycoplasma-positive here considered as negative sample (*n* = 10), were tested using single-target single-run and multitarget (TaqMan multitarget and SYBR Green multitarget) RT- qPCR in parallel. The threshold cycle (Ct) values of each individual run were compared with the Ct obtained at multitarget runs. The reproducibility of the multitarget RT-qPCRs was recorded by three independent runs. The sensitivity and specificity of the applied multitarget RT-qPCRs tested in the reference samples were verified, and true-positive and negative results were calculated using the web-based tool (https://www.medcalc.org/calc/diagnostic_test.php). The false-negative and false-positive results were verified using the positive and negative control within each sample panel.

## Results

### Outbreak investigations

A total of 364 poultry flocks, including layer, broiler and broiler breeder, Sonali, duck, pigeon, and turkey flocks, were investigated. Each type of bird has varied flock sizes (Table 1), and the vaccination schedule for respiratory pathogens was also varied, however, in most cases vaccination was not done. In general, the affected flocks reported clinical signs of off-feed, gasping, sneezing, coughing, drowsiness, decreasing output, and varying degree of mortality. Table 1 displays the outbreak overview for various bird species. The outbreak mostly occurred from pre- to post-winter seasons (October–May) in Bangladesh when the average temperature ranges from 15 to 30°C. At necropsy, the dead or affected birds showed congested and hemorrhagic trachea, cloudy air sac, hemorrhages in the intestine, brain, blackish, fibrinous, edematous, hemorrhagic, and congested and consolidated lung as shown in Supplementary Figure 1. Following the acquisition of respiratory clinical characteristics, samples from several commercial poultry flocks were examined using simultaneous RT-qPCRs.

**Table 1:** Summary of respiratory outbreaks in poultry in Bangladesh in the current study.

**Bird type**	**Number of flocks**	**Flock size range**	**Vaccination**	**Clinical signs**	**Mortality average**	**Major necropsy findings**
Layer	97	50–3,5000	Yes	gasping, sneezing, coughing, drowsiness, diarrhea, off feed, certain drop in egg production	3.6%	Congestion in trachea and lung; cloudy air sac, highly fragile liver, hemorrhages in cecal tonsil, intestine, pancreas, muscle, liver, brain, proventricular gland and gizzard
Broiler and Broiler breeder	115	100–12,500	Yes/No^a^	gasping, sneezing, coughing, ruffled feather, depression, off feed, certain drop in egg production (Breeder only)	7.3%	Hemorrhages in muscle, congestion in the trachea and lung, cloudy air sac, ascites; hydropericardium, fragile liver
Sonali	63	1,800–6,800	No	Respiratory distress, depression and off feed	5.8%	Congestion in the trachea, lung, cloudy air sac, liver, intestine and mesentery; mottled spleen
Duck	79	200–7,500	No	Most of them apparently healthy sometimes develop nervous disorder	2.5%	Congestion in the trachea and lung hemorrhages in the intestine and brain
Pigeon	8	20–50	No	respiratory sound, drowsiness, nervous signs, sudden death	35%	Congestion in the trachea and lungs, mottled spleen, hemorrhages in the intestine and brain
Turkey	2	15–30	No	respiratory distress, depression and off feed	2%	Congestion in the trachea and lungs; hemorrhages in the intestine

^a^Small-sized broiler flocks were mostly unvaccinated but bigger flock sizes and breeders were vaccinated properly.

### Application of RT-qPCR-based simultaneous assay

Two multitarget parallel RT-qPCR assays (TaqMan multitarget and SYBR Green multitarget) were applied, which were originally based on the “Riems Influenza a Typing Array” (34, 35). Each multitarget parallel assay's PCR plate or strip (depending on the number of samples to be analyzed in each RT-qPCR) was set so that each column can include six unknown samples, one positive and one negative control (Supplementary Table 2).

**Figure 1:**
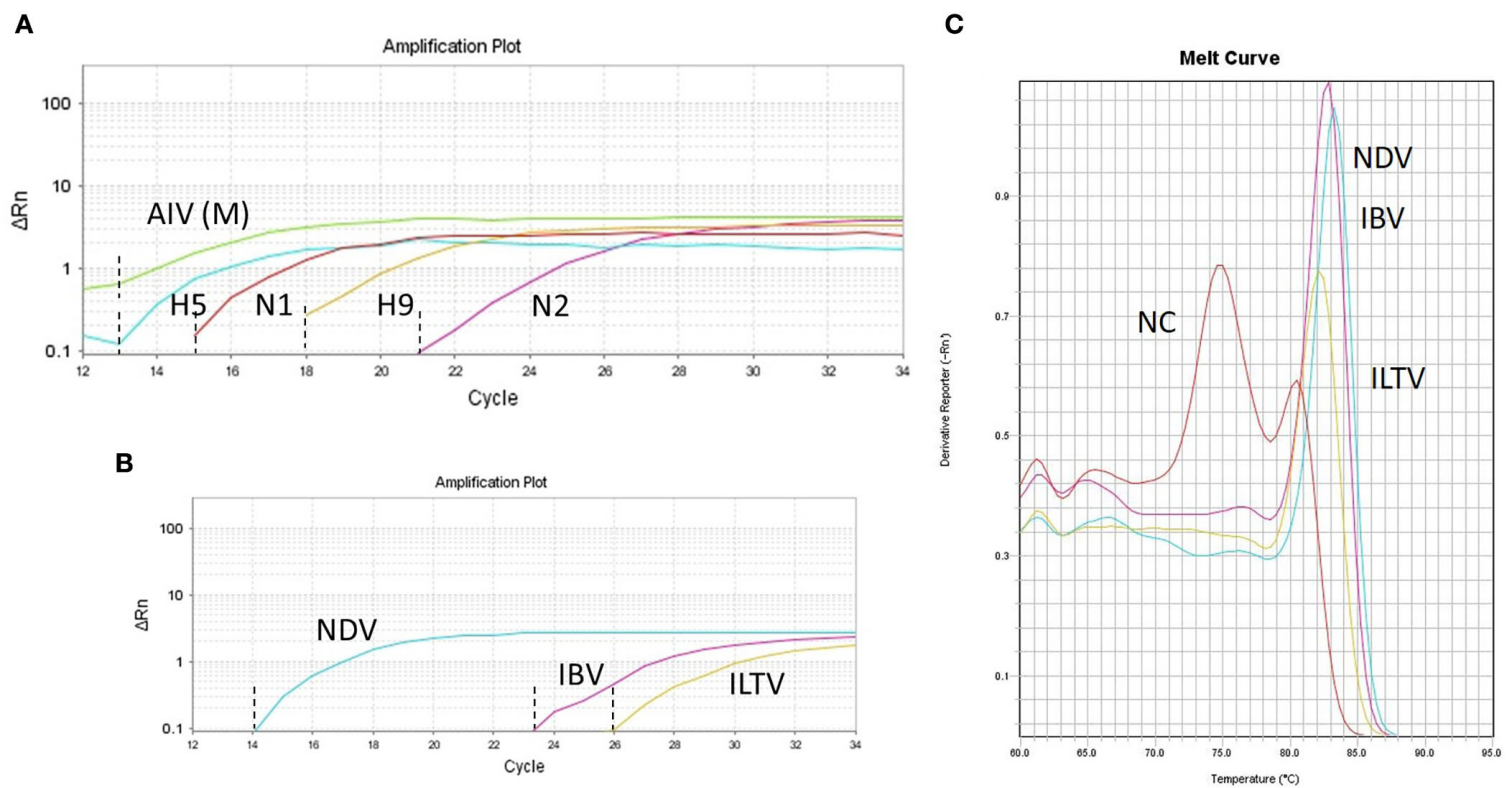
RT-qPCRs amplification plots for specific target viral pathogens. **(A)** Simultaneous detection of generic avian influenza virus (AIV-M) and subtyping of AIV (H5, H9, N1, and N2) in a single run. **(B)** Simultaneous detection of Newcastle disease virus (ND), infectious bronchitis virus (IBV), and infectious laryngotracheitis virus (ILT) along with the melt curve **(C)** in a single run. NC denotes a negative control that shows a melt curve at a different temperature (~75°C). The Ct values of individual gene is indicated by dash lines **(A,B)** generated from the number of cycles.

As all target viruses have RNA genome except ILTV (DNA genome), we applied RT-qPCR methods for SYBR Green multitarget parallel assay to facilitate simultaneous run. TaqMan multitarget RT-qPCR exclusively amplified the M, H5, H9, N1, and N2 genes of AIV in a single run (Figure 1A). Other respiratory viruses, such as NDV, IBV, and ILTV, were detected as well using SYBR Green multitarget RT-qPCR but in a separate run (Figure 1B). The construction of a dissociation melt curve (Tm) in SYBR Green multitarget RT-qPCR at temperatures between 83 and 84°C further aided the accurate identification of specific target viruses as the negative control produced an unspecific Tm at 75°C (Figure 1C). The final interpretation for multitarget RT-qPCRs was based on the Ct (cycle threshold) values on the sigmoid curve and the Tm values on the melt curve (SYBR Green multitarget only), and it was fairly good for detecting pathogen target genes (Figure 1). Ct values above 35 were considered for negative interpretation of the target pathogens.

### Comparison of viral load in single and simultaneous RT-qPCR

**Figure 2:**
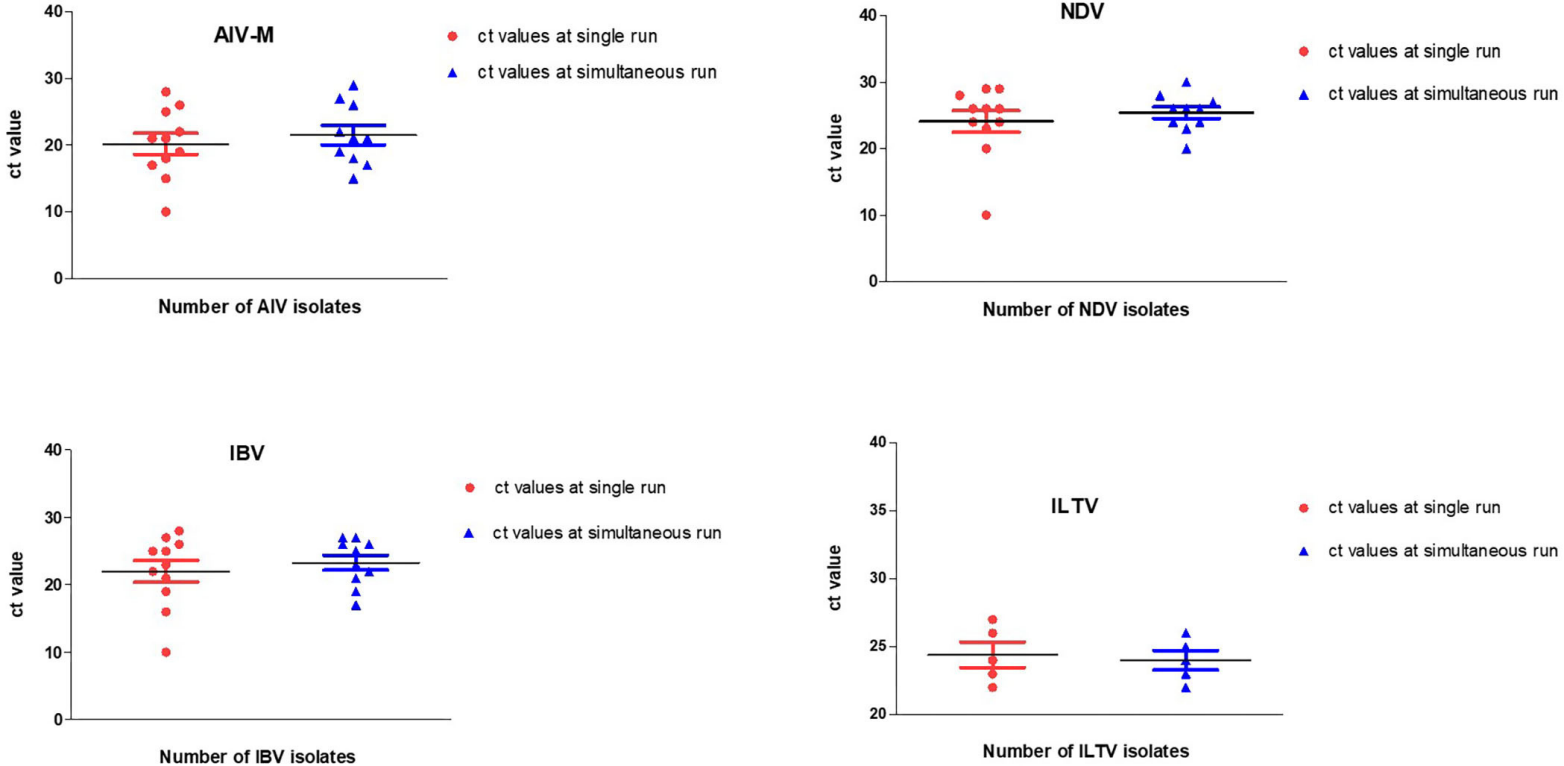
Scatter plots representing Ct values at two independent RT-qPCR runs. There were no significant variations in Ct values generated using single-target single run and multitarget parallel RT-qPCR simultaneous run.

All reference (positive and negative) viruses (*n* = 45) were tested blindly against all target genes using multitarget parallel RT-qPCR assays and compared with single-target RT-qPCRs. The resulting Ct and Tm values in specific targets at positive controls were then used as a baseline for the evaluation of other samples. Triplicate runs of the selected reference samples were employed in both single-target and multitarget parallel RT-qPCR assays. Each test performed using single- or multiple-target assays in two different thermal cyclers on two different days yielded almost similar Ct values (Figure 2). The mean Ct value for each target in two independent assays did not differ considerably, indicating that the applied simultaneous TaqMan and SYBR Green multitarget detection approaches are as specific as single-target RT-qPCR runs.

The semi-quantitation and detection limits for the multitarget simultaneous assays are given in Table 2. The applied TaqMan multitarget and SYBR Green multitarget simultaneous RT-qPCR assays maintained linearity of magnitude and, using the slope from the linear equation, the overall efficiency was estimated to be 99% as of single-target single RT-qPCR run. The three replicates also do not differ with the generation of Ct values and thereafter have shown the less standard error of the average Ct values (Table 2).

**Table 2:** Detection limit (Ct) and reproducibility of reference samples in applied multitarget simultaneous RT-qPCR assays.

**Reference samples**	**Single target RT-qPCRs**		**Multitarget simultaneous RT-qPCRs**	
	**Average Ct^**a**^**	**S.E.M^**b**^**	**Average Ct^**a**^**	**S.E.M^**b**^**
AIV (1–10)	21.2	±0.13	21.5	±0.12
NDV (1–10)	25.5	±0.24	25.4	±0.27
IBV (1–10)	23.2	±0.18	23.3	±0.15
ILTV (1–5)	24.4	±0.19	24.0	±0.18
Negative^c^ (1–10)	Undetermined	N/A	Undetermined	N/A

a Average Ct value from triplicate independent single-target and multitarget simultaneous assay runs.

b Standard error of the average Ct values.

c Known negative for AIV, NDV, IBV, and ILTV.

### Detection of target respiratory pathogens

**Figure 3:**
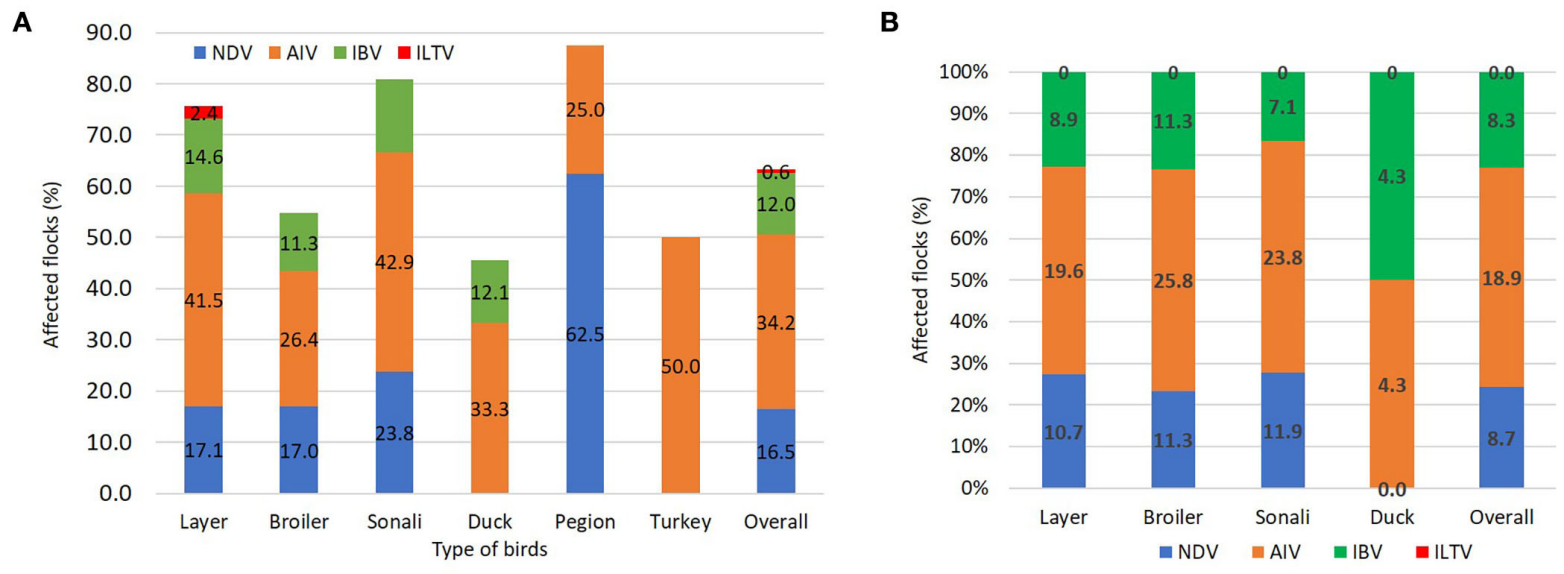
Detection of respiratory pathogens by applying the multitarget simultaneous RT-qPCRs. **(A)** The stacked column showing the percentage of respiratory pathogens in tissue samples among different bird types. **(B)** The stacked column showing the percentage of respiratory pathogens in oropharyngeal swab samples among different bird types.

Among the 158 tissue samples, there were 26 positives (16.5%) for NDV, 54 positives (34.2%) for AIV, 19 positives (12%) for IBV, and only a single positive (0.8%) for ILTV. On the other hand, 206 oropharyngeal swabs were detected 18 NDV (8.7%), 39 AIV (18.9%), and 17 IBV (8.3%) with no ILTV detection. Figure 3 shows the percentage of flocks afflicted by viral infections by bird type from tissue (Figure 3A) and swab (Figure 3B) samples.

**Figure 4:**
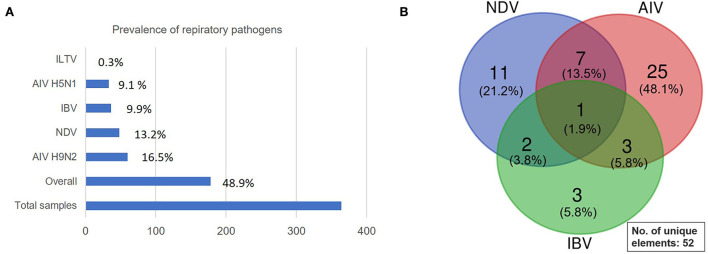
Overall prevalence of respiratory pathogens. **(A)** A clustered bar showing the overall prevalence of respiratory pathogens identified in the current study. **(B)** Venn diagram showing the presence of co-infections of 2–3 different respiratory viruses detected by multitarget simultaneous RT-qPCR assays.

Altogether, 48.9% of samples carried respiratory infections of which the highest detected (16.5%) pathogen was low pathogenic avian influenza (LPAI) subtype H9N2, followed by NDV (13.2%), IBV (9.9%), highly pathogenic avian influenza (HPAI) subtype H5N1 (9.1%) with only 0.3% ILTV (Figure 4A). Thus, within the AIV, 35% belonged to the HPAI subtype H5N1 and 65% were LPAI subtype H9N2. In addition, mixed viral infection was detected using the multiplex simultaneous RT-qPCRs, and the positive virus co-infection was found within the AIV, NDV, and IBV as depicted in Figure 4B. Briefly, 13 samples carried co-infections of which 7 were positives for both AIV and NDV, 2 positives for NDV and IBV, 3 positives for AIV and IBV while a single flock was positive for triple infection with AIV H9N2, NDV, and IBV. The 13 positive co-infected samples were further reconfirmed using single RT-qPCRs for each target independently, yielding almost identical amplification results.

## Discussion

The ability to detect and differentiate causative viruses from surveillance or outbreak samples quickly helps in controlling the spread of viruses. The current study focuses on the detection of respiratory viral infections in poultry and the circulation of either high or low pathogenic subtypes of AIV in Bangladesh. Another important aim is the application of rapid and sensitive real-time PCR-based multitarget simultaneous assays (TaqMan multitarget and SYBR Green multitarget) for the successful detection of respiratory viral pathogens. In this study, we used tissue (pooled lung and trachea) and oropharyngeal swab samples from dead or sick animals when flock morbidity and mortality were noticed. The detection and identification of avian respiratory pathogens, particularly AIV, IBV, and NDV, have been the subject of numerous research studies due to the significance of these viruses in commercial poultry.

Riems influenza a typing array (RITA) is an RT-qPCR-based low-density array diagnostic approach for the detection of 14 HA and 9 NA subtypes of AIVs (34), and an improved version of this method has been developed recently (35). The assay uses a hydrolysis probe technique (TaqMan), one of the most powerful and widespread methodologies in diagnostic microbiology (40). The RITA represents a competitive, fast, and sensitive subtyping tool that requires neither new machinery nor additional training of staff in a lab where RT-qPCR is already established (34). Researchers have used PCR extensively as an indispensable diagnostic technology to detect viruses since its introduction because of its high sensitivity and specificity. Based on this assay, we applied two multitarget simultaneous assays, one is TaqMan probe-based RT-qPCR for AIV detection and subtyping in a single run. The sample gives positive results against the target generic gene of AIV (M) and simultaneously detects the AIV subtypes (H5, H9, N1, and N2) within a short period of time. Multiplex PCR is the simultaneous detection of more than one virus in a single-tube reaction, whereas our approach is the uniplex PCR in multiwell layout. Due to the endemic nature of H5N1 and H9N2 in Bangladesh (19), the current study concentrated on subtyping these viruses. Recently circulated global havoc of the HPAI H5N8 subtype is not yet detected in Bangladesh; therefore, it is opted out of current multitarget assays. However, it is quite possible to incorporate N8 detection into the same TaqMan multitarget assays to detect the N8 subtype simultaneously in a future study. Another assay is SYBR Green-based RT-qPCR that can detect target genes against three other respiratory pathogens (NDV, IBV, and ILT) simultaneously in a single run. Such molecular approaches have been employed in the past with various target pathogen combinations and have proven to be highly useful (34, 39, 41).

Before applying the assays in field samples, we evaluated the assays with reference (*n* = 45) samples from the laboratory repositories. As those target primers have already been established and sensitivity and specificity were evaluated, sensitized (34, 37–39), and are using for many years, we further combined those targets as per the requirement of the current study. Further specificity of the multitarget assays was ensured when negative commercial flocks were turned out negative by applying the simultaneous RT-qPCR assays. The most commonly determined respiratory diseases in different poultry farms during the study period were avian influenza (AI), with a prevalence of 25.5% (HPAI H5N1 and LPAI H9N2), followed by Newcastle disease (ND) and infectious bronchitis (IB), each with a prevalence of 13.2 and 9.9%, respectively. ILT, on the other hand, was identified only in a layer flock, indicating an occasional infection. In the current study, AI and ND offered considerable challenges in the layer, broiler, Sonali, and pigeon flocks, whereas AI and IB had a significant impact on duck flocks. The death of the two turkeys examined here may not have been caused by any of the viral diseases tested here; however, one turkey was found to be positive for the LPAI subtype H9N2. These similar fundamental respiratory viral diseases have been identified previously in many other countries in Asia and Africa, including Bangladesh (4, 24, 31–33, 42, 43). For many years, Bangladesh has reported ~30–50% prevalence of respiratory infections in commercial poultry (22, 23, 42, 43), also in line with our observation, which has led to slow growth and productivity, generating substantial economic losses in the poultry industry. The overall mortality shown in the investigated flocks, however, may not solely be the result of the following four viral infections. Other co-infections and poor biosecurity might have accelerated the infectivity and death rate (41, 44, 45). In the current investigation, using the multitarget simultaneous RT-qPCR assays a total of 13 farms successfully detected co-infections of either AIV and NDV, IBV and NDV, or AIV and IBV, as well as a triple infection with AIV H9N2, NDV, and IBV.

Despite the fact that all the flocks evaluated in this study were suffering from respiratory diseases, none of them tested for *Mycoplasma gallisepticum* (MG) infections. The gross pathological observation, on the other hand, suggested that there was a good chance of obtaining MG-positive pathogens alone or in combination with other detected viral pathogens. As a result, a new investigation is needed to detect mycoplasma and other bacterial infections as a single or mixed pathogen. According to our current investigation, respiratory disease outbreak is one of the most challenging problems in the poultry sector of Bangladesh, which could be due to minimum biosecurity practices in the farms and inadequate vaccination programs. For the reduction of respiratory diseases in poultry, rapid and sensitive detection is required. Continuous monitoring of circulating viruses and reviewing farm vaccination programs using vaccines similar to the circulating strains in addition to effective vaccination are the strategies that need to be implemented.

## Conclusion

Respiratory pathogens are a major threat to the poultry industry in Bangladesh. AIV (HPAI H5N1 and LPAI H9N2), NDV, and IBV are the common respiratory pathogens circulating among poultry flocks. The TaqMan and SYBR Green multitarget simultaneous RT-qPCRs assays were successfully employed for screening the outbreak samples (tissue and swabs), and the interpretation was accurate, rapid, and reproducible. The specific laboratories having RT-PCR facilities could easily adopt the assays for simultaneous detection of respiratory viral pathogens within a shorter period of time as well as be able to do selective subtyping for AIVs that are endemic in the respective countries.

## Data availability statement

The original contributions presented in the study are included in the article/Supplementary material, further inquiries can be directed to the corresponding author.

## Ethics statement

The current study has been approved by the Ethical Committee of Bangladesh Agricultural University Research System under the approval number BAURES/ESRC /ET_28122. Written informed consent was obtained from the owners for the participation of their animals in this study.

## Author contributions

RP and CK wrote the first draft of the manuscript. RP, CK, IH, and AH organized samples and data collection. RP, CK, IH, AH, JB, MN, and MI analyzed formally and interpreted the results. RP, CK, IH, AH, JB, MN, MI, and EC edited and critically reviewed the manuscript. RP and EC designed the study and supervised the study. All authors have read and agreed to the published version of the manuscript.
