# Supplementary material for: Investigation of respiratory disease outbreaks of poultry in Bangladesh using two real-time PCR-based simultaneous detection assays
Source: Front Vet Sci. 2022 Dec 13;9:1036757. doi: 10.3389/fvets.2022.1036757 (PMC9792859; doi:10.3389/fvets.2022.1036757)
Supplement: Supplementary file 1 [file Data_Sheet_1.docx]

Supplementary Material

# Supplementary Table S1: List of avian viruses tested in this study with the used target primer and detection methods

| **Viruses** | **Assay** | **Primes and probes** | **References** |
| --- | --- | --- | --- |
| Avian influenza virus (AIV)  M gene | Probe based RT-qPCR | IVA-M1-F- AGA TGA GTC TTC TAA CCG AGG TCG  IVA-M1.1-R- TGC AAA AAC ATC TTC AAG TYT CTG  IVA-M1.2-R- TGC AAA GAC ACT TTC CAG TCT CTG  IVA-M1-FAM- TCA GGC CCC CTC AAA GCC GA | Spackman et al., 2003 |
| AIV  H5 gene | Probe based RT-qPCR | H5-F2-GTT CCC TAG YAY TGG CAA TCA T  H5-FAM2 FAM-CTG GTC TAT YYT TRT GGA TGT GCT CC-BHQ1  H5-R2- AAT TCT ARA TGC AAA TTC TGC AYT G | Hassan et al., 2022 |
| AIV  H9 gene | Probe based RT-qPCR | H9-F1- CAA TGG GGT TYG CTG CCT  H9-F2-CAA TGG GRK TTG CTG CCT  H9-FAM-TTY TGG GCC ATG TCI AAT GGR TC-BHQ1  H9-R-TTA TAT ACA RAT GTT GCA YCT G | Hassan et al., 2022 |
| AIV  N1 gene | Probe based RT-qPCR | N1-F- GRC CTT GYT TCT GGG TKG A  N1-FAM-CAA TYT GGA CYA GTG GRA GYA GCA T-BHQ1  N1-R- ACC GTC TGG CCA AGA CCA | Hassan et al., 2022 |
| AIV  N2 gene | Probe based RT-qPCR | N2-F1- AGTC TGG TGG ACY TCA AAY AG  N2-F2- CAG AGT RTG GTG GAC ITC  N2-FAM-CAT CAG GCC ATG AGC CTG TYC CAT-BHQ1  N2-R-TTG CGA AAG CTT AYA TNG VCA T | Hassan et al., 2022 |
| Avian paramyxo virus 1  (APMV-1) | SYBR-Green based RT-qPCR | APMV NP F: TGACAGYGACCAGATGAGCTT  APMV NP R: CCTGAGCCTGAGCRTACTC | Wäckerlin 2009 |
| Avian corona virus  (AvCov) | SYBR-Green based qRT-PCR | IBV_GU391: GCT TTT GAG CCT AGC GTT  IBV_GL533: GCC ATG TTG TCA CTG TCT ATT  Av-CoV-F: GGT TGG GAT TAT CCW AAR TGT G  Av-CoV-R: TGY TGT GAR CAA AAY TCR TG | Chaming et al., 2018 |
| Infectious Laryngotracheitis virus (ILT)  glycoprotein L gene | SYBR-Green based qPCR | ILTVgCU771-CCT TGC GTT TGA ATT TTT CTG T  ILTVgCL873-TTC GTG GGT TAG AGG TCT GT | Callison et al., 2007 |

# Supplementary Table S2: Plate set up for the multitarget simultaneous RT-qPCR assays. a) Plate set up for TaqMan multitarget RT-qPCR for detection of AIV M, H5, H9 N1 and N2 genes. b) Plate set up for SYBRGreen multitarget RT-qPCR for the detection of target gene of NDV, IBV and ILTV.

a)

|  | **AIV-M** | **H5** | **H9** | **N1** | **N2** |
| --- | --- | --- | --- | --- | --- |
| A | S1 | S1 | S1 | S1 | S1 |
| B | S2 | S2 | S2 | S2 | S2 |
| C | S3 | S3 | S3 | S3 | S3 |
| D | S4 | S4 | S4 | S4 | S4 |
| E | S5 | S5 | S5 | S5 | S5 |
| F | S6 | S6 | S6 | S6 | S6 |
| G | PC | PC | PC | PC | PC |
| H | NTC | NTC | NTC | NTC | NTC |

b)

|  | **NDV** | **IBV** | **ILT** |
| --- | --- | --- | --- |
| A | S1 | S1 | S1 |
| B | S2 | S2 | S2 |
| C | S3 | S3 | S3 |
| D | S4 | S4 | S4 |
| E | S5 | S5 | S5 |
| F | S6 | S6 | S6 |
| G | PC | PC | PC |
| H | NTC | NTC | NTC |

*S (S1-S6) represents sample number; PC: positive control; NTC: no template control; A-H: PCR plate or strip marking at first column where A-F can be used for unknown sample and G & H used for PC and NTC respectively.

**3. Supplemental Figure S1:**


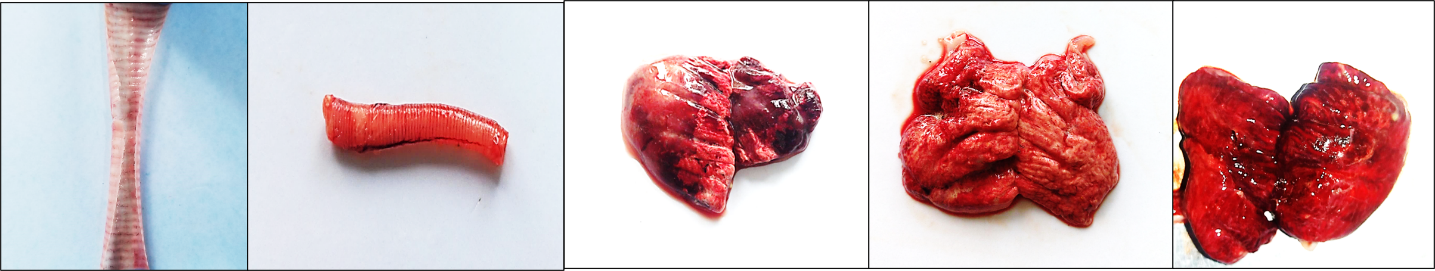


**Supplemental Figure S1:** Gross pathology of the respiratory organs (trachea and lung) identified in the examined birds. From left to right, hemorrhages in the trachea, congested trachea, consolidated and edematous lung, and mild to moderately congested lung.
